# Concurrent Infection with Dengue and Malaria: An Unusual Presentation

**DOI:** 10.1155/2013/520181

**Published:** 2013-03-30

**Authors:** Mohsin Bin Mushtaq, Mehmood I. Qadri, Aaliya Rashid

**Affiliations:** ^1^Sher-e-Kashmir Institute of Medical Sciences, Medical College Hospital, Srinagar 190002, India; ^2^Al-Kabir Medical Center, Srinagar 190002, India

## Abstract

Dengue and malaria are both endemic in South Asia and represent a major public health burden in this region. Though there have been some case reports of concurrent infection with dengue and malaria, yet there are only few cases of such infections reported from South Asia. Here, we present a case of a young male returning from a dengue endemic area who tested positive for the virus along with *Plasmodium vivax* and *Plasmodium falciparum*. In view of the severity of coinfection (Epelboin et al., 2012), overlapping symptoms, and a challenging obscurity of diagnosis, a multidimensional diagnostic approach is suggested.

## 1. Case History

 A 25-year-old male, resident of Srinagar, businessman by occupation returned to Srinagar after a weeklong trip to Delhi in October 2012. The patient started developing fever, chills, myalgias, and headache two days following his return and self-treated himself with Paracetamol. Prophylactic treatment for malaria was not given either before or during the trip. The patient subsequently developed severe headache and high grade fever of 102°F and was admitted to our hospital. Physical examination was unremarkable and haemogram revealed a normal study except for a low platelet count of 86,000/mm^3^. His renal and liver function tests, spot urine examination, chest X-ray, and USG abdomen were ordered and all parameters were within normal limits. Since the patient had visited Delhi during an outbreak of dengue and was found to have a low platelet count, a viral serology test using IgM ELISA for dengue virus was carried out that turned out to be positive. A diagnosis of dengue fever was made and supportive therapy was initiated. Patient continued to have fever spikes and chills despite the therapy. Since Delhi is also endemic for malaria, rapid card test for malaria along with thick and thin blood smears for malarial parasite was also carried out. The Malaria Ag (pLDH/HRP2) card test was positive for both *Plasmodium vivax* as well as *Plasmodium falciparum* and blood smears showed ring forms of *Plasmodium falciparum*. This suggested that the patient had concurrent dengue fever and malaria. The tests for Malaria were repeated to ensure conformity and the tests were again positive for both *Plasmodium vivax* and *Plasmodium falciparum*. The patient was put on 600 mg of oral Chloroquine Phosphate followed by 300 mg after six hours and then 300 mg daily for two days. Patient responded well to the treatment. Fever subsided and his general condition improved. He was also put on radical cure for malaria with 30 mg of Primaquine daily for 14 days (after his G6PD levels returned to normal).

## 2. Discussion

Concurrent infection with two different infective agents leads to an overlap of their clinical features that can pose a diagnostic challenge to the physician, especially in endemic areas. In addition, recent studies indicate that a coinfection may be more severe [1].

Earlier there have been reports of concurrent infection of dengue virus with a flavivirus, Chikungunya [2] and with different bacteria including *Salmonella Typhi* [3], *Shingella Sonnei* [4], and *Leptospira* spp. [5]. The first case of concurrent dengue and *Plasmodium falciparum* was published by Charrel et al. in 2005 [6] where the concurrent infection was diagnosed in a patient returning to France after 18-day travel to Guinea, Senegal, and Sierra Leone. It was followed by a case report of concurrent dengue with *Plasmodium vivax* in 2006 [7] while Bhalla et al. reported the first case from India in 2006 [8]. To the best of our knowledge, this is the second paper from India of mixed infection of dengue virus with two species of malarial parasite (*Plasmodium falciparum* and *Plasmodium vivax*). The first case was reported by Kaushik et al. in 2007 [9]. In addition, a study conducted in 2010 by Santos Santana et al. in the Amazon region revealed presence of dengue virus serotype 2 along with active *Plasmodium vivax* infection in two serum samples out of the total 111 samples investigated [10]. Studies have also revealed the high prevalence of mixed species malaria infection. In 2008, a study conducted by Lorenzetti et al., whereby DNA was extracted from 115 thick blood film *P*. *Falciparum *human blood positive samples, revealed that seventy-three percent were of *P*. *falciparum* single infections and 26.95% of mixed infections [11].

The accuracy of a serological test to diagnose dengue in patients experiencing malaria has been questioned earlier because reactivity is often nonspecific on certain rapid serological assays [12]; however, IgM ELISA serological test has demonstrated more than 90% specificity for dengue. The Malaria Ag (pLDH/HRP2 (Histidine rich protein)) card test for *Plasmodium falciparum* and *Plasmodium vivax* was used twice to ensure confirmation. Earlier analysis by Bharti et al. revealed that overall the rapid diagnostic card test for *Plasmodium falciparum* and *Plasmodium vivax* was 93% sensitive and 85% specific with a positive predictive value (PPV) of 79% and a negative predictive value (NPV) of 95% [13].

According to Carme et al. in French Guiana the specific rate of concurrent malaria and dengue infection from overall febrile patients was equal to 0.99 [14], which indicates that there is high chance of concurrent infection in that setting. It would be expected therefore that since both infections are endemic in our area, coexisting malaria and dengue infection could be common. However, there is little published evidence of such dual malaria and dengue infections despite both diseases being coendemic in South Asian region. Malaria and dengue are difficult to differentiate clinically as is emphasized by this case, yet the treatment of the illnesses is different and delay in appropriate therapy can be devastating, especially in malaria [15]. Endemic areas of malaria and dengue overlap to a large extend in South Asia and acquisition of both mosquito-borne infections concurrently is quite possible. We suggest that such concurrent infections should always be kept in mind by the physician while encountering such clinical situations as such mixed infections are likely to occur more frequently than reported in the available literature.
